# Hospital-based health technology assessment (HTA) in Finland: a case study on collaboration between hospitals and the national HTA unit

**DOI:** 10.1186/s12961-016-0095-2

**Published:** 2016-04-05

**Authors:** Esa Halmesmäki, Iris Pasternack, Risto Roine

**Affiliations:** The Hospital District of Helsinki and Uusimaa (HUS), PO Box 705, 00029 Hus, Finland

**Keywords:** Case study, Collaboration, Evaluation, Health technology assessment, Hospitals, Hospital-based health technology assessment, Technology

## Abstract

**Background:**

This study examines, as a part of the European Union funded Adopting Hospital Based Health Technology Assessment (AdHopHTA) project, the results and barriers of collaboration between Finnish hospitals and the national health technology assessment (HTA) agency, Finohta. A joint collaborative HTA program has existed since 2006 between the Finnish hospitals and the national agency.

**Methods:**

A case study method was used. Information about the collaboration between Finnish hospitals and Finohta was retrieved from interviews and publications, and categorised per theme. Hypotheses and indicators of successful collaboration were determined beforehand and reflected on the observations from the interviews and literature.

**Results:**

Overall, 48 collaborative HTA reports have been performed during 7 years of collaboration. However, there were no clear indications that the use of HTA information or the transparency of decision-making regarding new technologies would have increased in hospitals. The managerial commitment to incorporate HTAs into the decision-making processes in hospitals was still low. The quality of the collaborative HTA reports was considered good, but their applicability in the hospital setting limited. There were differing expectations about the timing and relevance of the content. Signs of role conflict and mistrust were observed.

**Conclusions:**

Despite collaborative efforts to produce HTAs for hospitals, the impact of HTA information on hospital decision-making appears to remain low. The difficulties identified in this case study, such as lack of managerial commitment in hospitals, can hopefully be better addressed in the future with the guidance and tools having been developed in the AdHopHTA project. Collaboration between hospitals and national HTA agencies remains important for the efficient sharing of skills and resources.

## Background

WHO defines health technology as the application of organized knowledge and skills in the form of medicines, medical devices, vaccines, procedures and systems developed to solve a health problem and improve quality of life. Health Technology Assessment (HTA) is research-based, practice-oriented evaluation of relevant available knowledge on both the direct and intended, as well as the indirect and unintended, consequences of health technologies [1], in the short and long term [2]. The consequences include clinical benefits and economic and organizational impact, as well as the social, ethical and legal implications associated with the health technology being assessed. The aim of HTA is to provide responses to the specific questions asked by the decision-makers on the likely value of health technologies. Methodological rigour and inclusiveness are required when collecting and analysing context-specific information for an HTA report. HTA is generally performed in national or regional HTA agencies or units. There are currently 54 members from 33 countries around the globe in the International Network of Agencies for Health Technology Assessment (INAHTA) [3]. A European network, the EUnetHTA, supports collaboration between European HTA organizations.

HTA performed in the hospital context is termed hospital-based health technology assessment (HB-HTA). Although it is usually performed in hospitals, it can also sometimes be outsourced to independent research bodies. HB-HTA provides responses to hospital managers’ questions relating to implementation of new technologies in their hospitals. Hospitals are generally the entry point for many new technologies. Therefore, it is necessary that they have the capability to assess their usefulness in a scientifically valid manner. The new technologies may replace or add on to existing technologies, which means that decision-makers need to know their value in relation to the current standard practice in their hospital. Furthermore, the information needs to be in place when implementation decisions are made in the hospital, which means that the assessment timelines are usually strict.

The Adopting Hospital Based Health Technology Assessment (AdHopHTA) project 2012–2015 was a European Union (EU) funded project aiming at strengthening the production and use of HTA in the hospital setting. As HB- HTA utilises the same methods and resources as national HTA, collaboration is likely to be beneficial. One of AdHopHTA’s aims was to examine the existing interactions between HB- and national HTA functions in its partner countries, and the barriers and facilitators of fruitful collaboration. This case study describing the Finnish context is part of that exercise.

Finland is a small country with 5.4 million inhabitants. It has a National Health System funded through taxes levied by the municipalities. Primary care is currently mostly provided and arranged by the municipalities. For the organization of specialized healthcare, the country is divided into 20 hospital districts funded by the municipalities. One hospital district can run several hospitals. The largest of them, the Hospital District of Helsinki and Uusimaa, has 24 hospitals throughout the province of Uusimaa in southern Finland. Altogether, there are approximately 100 hospitals in the country. Some specialized medical care services (e.g. organ transplantations, treatment of severe burns, etc.) are organized on the basis of special responsibility areas of university hospitals of which there are five. The intention of the present government is, during the next few years, to integrate primary care, specialized care and social services together and divide the country into 15 districts responsible for the organization of those services.

**Fig. 1:**
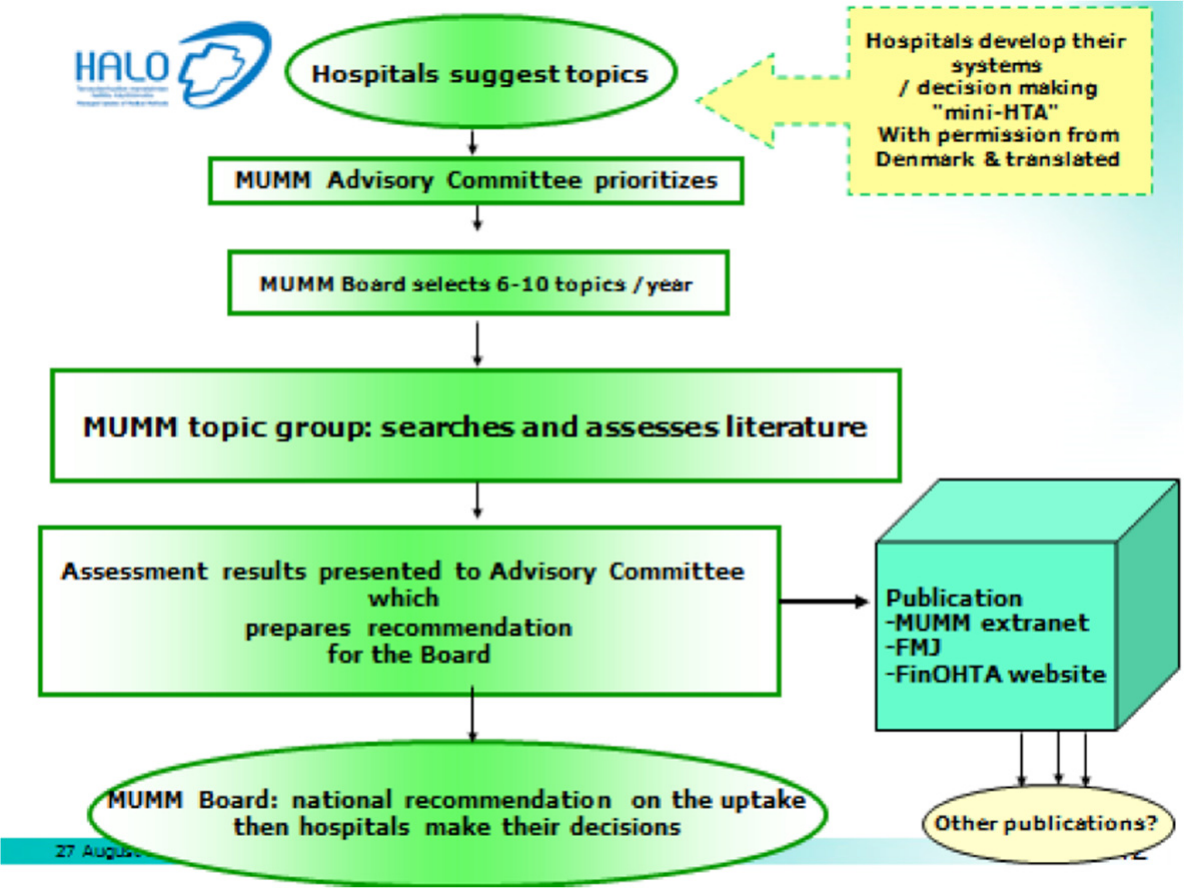
Process of Managed Uptake of Medical Methods programme – A joint program between Finnish national agency for health technology assessment (Finohta) and Finnish hospitals

The national Finnish HTA-agency, Finohta, established in 1995, is currently hosted by the National Institute of Health and Welfare. In 2013, there were approximately 20 people working for Finohta (not all full time). Since 2006, Finohta has collaborated with the 20 hospital districts of Finland in joint HTA production within the Managed Uptake of Medical Methods (MUMM) program (Fig. 1). The hospitals are responsible for topic identification as well as formulation and implementation of recommendations. Finohta carries the responsibility of coordination, and provides expertise on literature search and assessment methodology; the actual assessments are performed jointly [4].

There was very little HTA activity in Finnish hospitals prior to their involvement in the MUMM program in 2006. The first dedicated physician post for HTA-related work in a hospital was established in 2001 in Helsinki University Hospital. Two other university hospitals followed suit towards the end of the decade. A more rapid and hospital-centred approach using the mini-HTA form [5] was proposed in 2012, and has now been adopted by most of the five university hospital regions. Technologies requiring more thorough evaluation are still intended to go to the MUMM program.

This case study examines the content of the collaborative MUMM program after its 7 years of function. Furthermore, it reports how clinicians and healthcare decision-makers perceive the program and tries to identify the barriers and facilitators of success.

## Methods

This case study employed a multimethod approach and combined information from a reference search in Medline and a Finnish health portal (Terveysportti), 12 own interviews, and data from 38 interviews performed earlier by Dr Outi Simonen as part of her doctoral thesis on how the concept of effectiveness is understood by Finnish healthcare decision-makers [6]. Information concerning the MUMM program obtained from all the sources were grouped into the following themes: history of the collaboration, volume or intensity, content, coordination, impact on decision-making, perceived or observed benefits, and drivers and barriers of collaboration. Potential indicators of successful collaboration as well as other contextual factors possibly affecting collaboration between hospitals and HTA agencies were identified prospectively in discussions within an international team of researchers within the AdHopHTA project. Based on the information obtained from the above mentioned sources, hypotheses were developed about what the determinants of successful collaboration would be.

### Information sources

A PubMed search and 12 interviews were performed in May–June 2013. The informants were: four clinicians from Helsinki University Hospital who had been previously involved in MUMM; four current or former employees of Finohta who had been active in MUMM; three external stakeholders who had worked for, or otherwise had close connections with, Finohta; and one lead person from the HTA unit of the Finnish Medicines Agency (Fimea). Specific questions were used to stimulate the non-structured interviews. These were: What are the current interactions between hospitals and the national HTA agency? Is the collaboration beneficial? What are the barriers of good collaboration? How should the collaboration work? Interviews were recorded and their content grouped by themes in a report. The report was sent to the interviewee for validation: only minor corrections or amendments were requested. One of the informants provided us also with unpublished information from an internal review of the MUMM program based on register data and interviews performed in 2012. This information has later been published as an article in the Finnish Medical Journal [7]. Moreover, as mentioned above, data from 38 previously performed interviews by Outi Simonen in 2008 as part of her doctoral thesis were also utilised as they contained questions about knowledge of the MUMM program [6]. The interviewees of that study were 13 hospital managers, 12 chief physicians, and 13 nursing directors representing all five university hospital districts in Finland. Websites of Finnish hospitals and healthcare research institutions were screened for further relevant information.

### A priori considerations about success of collaboration

Contextual factors potentially affecting the success of collaboration between hospitals and national HTA units were identified by the authors in the general HTA literature on interactions observed in various types of collaborations [8–10]. Identified aspects included the power and role of HTA information in decision-making at the national level (including reimbursement and pricing) and at the hospital level (including clinical implementation and purchase); size of country; history and volume of HTA activities nationally and in hospitals; funding system of HTA activities (closed budgets vs. activity based); level of centralisation of decision-making in hospitals; and the spectrum of HTA topics and qualities of assessment methods and products. These were discussed in an international team of researchers within the AdHopHTA project and, based on these considerations, potential success indicators for collaboration were proposed and some hypotheses suggested. The applied indicators of successful collaboration are listed in Box 1. The hypothesised predictors of successful collaboration were an established HTA function, powerful HTA (which has legal mandate), fixed budgets for HTA function in hospitals, and sharing of individuals or competence (not only information and data). Furthermore, a hypothesis was made of a barrier for good collaboration, and this was the differing expectations of HTA by the hospital managers and clinicians on the one hand, and HTA professionals on the other [11].

**Box 1:** **Indicators of successful collaboration in health technology assessment (HTA) between hospitals and national HTA units, a priori defined for this study**

Collaboration increases the use of HTA in clinical implementation and purchase decisions in hospitals
Collaboration enhances transparency of decision-making regarding the implementation and purchase of technologies in hospitals
Collaboration enhances patient safety
Collaboration increases patients’ and the public’s acceptance of technologies and access to them
Collaboration enhances equity in the society (equal access to treatments)
Collaboration promotes innovation and clinical research

## Results

A PubMed search using the full name and acronym of MUMM gave no references and a search combining the search terms ‘HTA’, ‘hospital’ and ‘Finland’ yielded only one study [12]. The Finnish portal for healthcare professionals (Terveysportti), which contains information from the Finnish professional journals that are not Medline indexed, was consulted using the corresponding Finnish search terms, and resulted in 13 articles mentioning the MUMM program [12-24].

### History of collaboration

In the early years, the late 1990s and early 2000s, the national HTA agency, Finohta, provided methodological, and sometimes also financial support for research projects in hospitals, mainly for randomised trials and systematic reviews. The aim was to increase the critical mass of people able to conduct evaluation research in Finnish hospitals. According to some of the interviewees, this activity was considered useful by hospitals and Finohta but it was stopped due reorganisations and cost cuttings in the late 2000s.

Since the turn of the millennium, several articles were published in national professional journals about the importance of systematic evaluation of both new and obsolete healthcare technologies, particularly in hospitals. A structured approach to nationally coordinate the evaluation and uptake of new medical technologies was, according to the interviewees, clearly desired, especially by hospital decision-makers. Starting in 2004, a series of discussions began between hospitals on how to identify joint topics for assessment. The idea was to encourage the hospitals to systematically demand critically appraised information on effectiveness, safety and costs of new technologies, and make joint decisions concerning their uptake. This was considered necessary for reducing the geographical variation in the availability of new technologies and for securing rational use of resources. Models from other countries, such as Denmark and the United Kingdom, stimulated the development.

In September 2005, a group of hospital directors decided in their yearly meeting to establish collaboration between hospital districts and Finohta. The focus of the programme was set on new technologies. The kick-off meeting of the new collaboration was in December 2005. One of the initiators toured the 20 hospital districts of Finland in 2006 and 2007, promoting the new MUMM program and engaging the clinicians and hospital managers to its principles. It was clearly outlined from the beginning that MUMM is a joint venture of Finohta and the hospital districts. Almost all hospital districts agreed to join, or at least to follow-up on the progress of the program.

MUMM has been a priority function in Finohta and, despite resource restrictions, the input to MUMM-HTAs has remained relatively stable during the years. However, there is currently some pressure for Finohta to prioritise actions, and therefore some deliberations have been made to streamline the organisational structure of MUMM and even diminish MUMM-HTA production.

### Content of collaboration

The collaborative MUMM-HTA product is typically a 5- to 10-page document based on a systematic review of evidence on effectiveness and safety, and in some cases the purchasing and running costs of the new technology. The duration of the overall project, from topic selection to recommendation, is typically more than 1 year. The manuscripts of the MUMM-HTAs are submitted for peer review and published in the *Finnish Medical Journal*.

Hospitals select the topics, which include mainly medical devices and procedures. Pharmaceuticals were deliberately excluded because of resource constraints and because it was long anticipated that there would be another HTA function within Fimea to cover these later. This activity is currently being developed by Fimea.

A typical MUMM-HTA is drawn up by two clinical experts, usually physicians, from hospitals, and two HTA-specialists and an information specialist from Finohta. Finohta carries the responsibility of coordination of the MUMM-HTA projects. Hospital clinicians participate either as authors or expert consultants in the MUMM-HTAs. They are recruited specifically for each project, and their identification is based on individual networks and contacts of the Advisory Board and Finohta instead of a formal procedure for nomination.

After the MUMM-HTA has been completed, the hospital districts are responsible for formulation of the recommendations based on the review. The Advisory Board of MUMM, which consists of clinical experts from hospitals, prepares a preliminary recommendation which is then discussed and finalised by the MUMM Board, consisting of chief medical officers of the hospital districts.

Assessment and appraisal are clearly separated in the MUMM process. This means that the recommendations are drawn up by other people than those who performed the review; this has been considered a valid principle. It was even suggested that the recommendation phase should be taken out of the current MUMM framework and performed completely within the hospitals, incorporating it into the existing meeting and decision-making structures. This would probably promote the actual implementation of the recommendations in hospitals as it currently discretionary for each hospital to adopt the MUMM recommendations.

### Volume of collaboration

Presently, there are 51 MUMM reviews, based on which 56 recommendations have been given. Some of the reviews have led to two or three different recommendations for different indications. Resource input for MUMM has been greater from the side of Finohta than from the hospitals. During the first 7 years of collaboration, Finohta has contributed to the collaborative MUMM project a total of 4 to 5 person years and approximately €60,000 annually as additional direct costs. The hospital districts have overall provided 90 clinical expert inputs from 30 disciplines during the same time. The role of the clinical experts from hospitals is clearly part time, sometimes consultative, which means that most of the actual work in the assessment phase has been performed in Finohta.

### Impact on decision-making

In 2012, an internal evaluation of the MUMM program was performed using information from hospital registers and interviews of chief physicians. The aim was to evaluate the perceptions concerning MUMM and its impact on decision-making in hospitals. Results showed that 57% of chief physicians felt that the content of the MUMM recommendations are unambiguous, 28% felt that the recommendations are sufficiently communicated, and 29% stated that the recommendations are followed [24]. Hospital managers had a more positive attitude towards HTA activity than other professional groups in hospitals.

Hospitals are dependent on Finohta’s input in the MUMM-HTAs and would have difficulties in replacing the skills and resources provided by Finohta. On the other hand, the knowledge concerning MUMM in hospitals is still inadequate and the current MUMM-HTAs and recommendations are scarcely used in decision-making, which indicates that hospitals do not depend on the outputs of the collaboration. MUMM is a strategically important function for Finohta but not essential for its overall functions. There are other competing national HTA functions and tasks where the resources could be shifted if there was no MUMM.

According to most interviewees, the MUMM program seems to remain poorly known and underutilized in decision-making in hospitals. One obvious reason is that the results of MUMM reviews are not systematically required or used in the clinics’ purchase decisions.

The hospital clinicians pointed out the need for two-stepped decisions: conditional with the requirement for re-assessment when new evidence emerges, and final. MUMM recommendations were considered “*too vague*” to sufficiently guide decisions of conditional decisions. The requirements for data to be collected in the clinic should be detailed and the timeline agreed upon after which a reassessment is made before the final decision. Some clinics have their own systems to collect evidence and monitor the uptake of new technologies. Teaching clinics may perform evaluation research, e.g. randomised controlled trials and systematic reviews, on which to base their decisions. MUMM seems to be only one of the possible routes through which evidence is taken into decision-making in hospitals.

### Barriers of collaboration

HTA can still today be considered a threat to clinical autonomy according to the interviewed clinicians. According to some interviewees, clinics want to be “*frontline*” and compete with other clinics about reputation and staff. The original indications of a new technology may be narrow and it is tempting to use the technology also in less severe cases, although expanding indications at a certain point may cause more harm than good to the patients. Unnecessary interventions may also be performed for fear of claims, or because of extra earnings gained. All these issues, together with the constant budget competition between clinics, maintain the current situation where physicians are not willing to limit the uptake or use of new technologies. Furthermore, HTA, and consequently also MUMM, is considered mainly as a tool to restrict the uptake.

In order to increase the impact of MUMM in decision-making, hospital clinicians suggested that MUMM reviews and recommendations should be more regularly communicated to the clinic management level where the actual decisions are made. Currently, the information is distributed to the higher level in the hospital hierarchy, which is apparently not sufficient [25].

Signs of a role conflict and resulting mistrust occurring between hospitals and Finohta were also identified. Mistrust is reflected in expressions such as “*hospital clinicians do not feel that they belong to MUMM*” and “*Finohta isolates and wants to steer too much the collaboration*”. On the other side, Finohta staff felt that “*hospitals’ attitude is arrogant*” and stressed that “*Finohta is not willing to act only in a coordinating role, but have a say in the design and content of the program in the future too*.”

The slow assessment process is one of the main barriers of MUMM. As the technologies appear and evolve rapidly, the MUMM reviews should be quicker and more effort should be made in updating them regularly. An ideal time span for a MUMM review was originally set to 6–9 months. In the early years of the program this was considered a realistic target, but in recent years it has become apparent that this is not the case. A slow and thorough process may be justified, however, if the topic requires careful and multifaceted evaluation including ethical considerations. Nevertheless, for most topics, a quicker process was required; this is particularly claimed by hospitals but acknowledged by Finohta as well.

Several factors have led to the process being so slow. Clinicians participate through minor input, often on their own time. The staff of Finohta is occupied with several parallel tasks, and prioritisation has not always favoured MUMM. With the current resources, the already slow process of MUMM reviews is not going to be accelerated; on the contrary, it will most likely be even slower.

### Reflecting on the hypotheses

Collaboration between Finnish hospitals and the national HTA unit cannot be considered successful when looking at the indicators of success defined for this case study. The use of HTA information in hospital decision-making has not increased substantially. The transparency of the clinical implementation and purchase processes in hospitals is still poor, which also prevents exact evaluation of the impact of HTA. All other success indicators related to increased patient safety, equity and acceptability as well as innovation support, could not be observed in the interviews or published documents used for this case study.

One of our hypotheses was that powerful HTA, which has a legal mandate, predicts successful collaboration. In Finland, HTA has no power as there is no clear legal obligation to use evidence in decision-making nationally or in hospitals. This is probably one of the reasons why the collaboration between hospitals and the national HTA unit has not been particularly successful. Another hypothesis, which is supported by the case study in Finland, is that the differing expectations seemed to be a major barrier for collaboration.

Finally, it was hypothesised that collaboration between hospitals and HTA units would be deeper in countries with a long history of HTA. Finohta is one of the oldest HTA units in Europe, founded in 1995, but still the collaboration was not strikingly successful. It was anticipated that sharing people and knowledge strengthens the collaboration, but also this remained unverified by the results of this study. As mentioned above, Finland is a small country with 5.4 million inhabitants, the funding of HTA is based on state budget, and the decision-making structures regarding technologies for hospitals are extremely decentralised. Whether these features represent barriers of a successful collaboration needs to be confirmed by further studies.

## Discussion

According to our results, the MUMM program does not appear to have reached its full potential and is still poorly utilized in decision-making in hospitals. One explanation for that may be the fact that many hospitals are only starting to require evidence-based appraisal of scientific literature for their purchase decisions. Another one is certainly the slow assessment process. When considering new technologies, the clinicians would, in most cases, like to have the evidence instantly, they are not willing to wait for a year.

The gap between research and practice or policy has also been addressed in earlier studies. A recent systematic review identified [26], as the most frequently reported barriers to evidence uptake, poor access to good quality relevant research and lack of timely research output. Facilitators identified were similar to those identified in our study, namely collaboration between researchers and policymakers, and improved relationships and skills. In Denmark, an information campaign to introduce mini-HTA as a decision support tool for the municipalities was reported to be insufficient, and should have, according to the authors, been supplemented with a strategy to secure local political/managerial support and willingness [27]. Implementation of HTA appears thus not just to be a question of how to increase the use of evidence in decision-making, but as a matter of reforming local decision processes [27].

What could be done to overcome the barriers? One way forward could perhaps be to attempt to address the needs of decision-makers more directly, e.g. by adding information on budget impact and organisational issues. The results of the AdHopHTA project also highlight other information needs of hospital managers, namely those related to the strategic and political importance of the decisions [28]. Comparative national or international information regarding treatment indications, clinical outcomes and costs would be essential particularly when expensive technologies are considered, and would allow planning of resource allocation on a national level. Rapid assessment of most new technologies might be more helpful for decision-makers than a thorough evaluation of only some. Rapid assessments downloaded into an easy-to-use common database could be an improvement despite of the critique concerning their thoroughness. Hospitals should learn to utilize the work of others and study the geographical differences in treatment indications, clinical outcomes and costs in order to identify the factors that predict most successful function. For that purpose a centralised function producing information on treatment outcomes and indications is warranted. Currently, the National Institute for Health and Welfare generates some of the information needed, but an even volume would be desirable.

Terminology is important. The concepts of HTA, MUMM and mini-HTA are taken to the hospitals from the world of HTA. They do not relate to anything familiar for clinicians and may therefore cause natural resistance. In some countries, attempts have been made to rename HTA-functions; this could also be worth trying in Finland.

From a hospital point of view, there is no need to keep the assessment of pharmaceuticals and non-pharmaceuticals as separate lines of activities, one involving Finohta and the other Fimea. Although pharmaceuticals need to demonstrate efficacy and safety before market access, the process of their uptake in hospitals is not clearly different from that of devices and procedures where less evidence requirements exist [13].

Collaboration in clinical trials could be an area of co-operation which could be restarted. The MUMM reviews are able to identify the areas where evidence is scarce and this information could systematically feed into the research agendas in hospitals. Some of difficulties identified in this case study, such as lack of managerial commitment in hospitals, can hopefully be better addressed in the future with the guidance and tools being developed in the AdHopHTA project. Research funds for hospitals have decreased substantially. It would, therefore, be particularly important to target the research to fill in the gaps identified in evidence of effectiveness and safety of hospital interventions. Reduced clinical research budgets may also lead to a situation where more and more technologies are taken directly into use without evidence of their real life effectiveness.

## Conclusions

Collaboration between hospitals and national HTA units is in principal supported in Finland, but the current program, in the current societal situation without any clear legal obligation to use evidence in decision-making, has not gained sufficient support after the first 7 years of existence. The most obvious barriers for collaboration have been the lack of managerial commitment in hospitals, lacking timeliness of the assessments and a role conflict between the collaborating parties. Moreover, HTA is not the only route to take evidence into hospital decisions: activities and structures for this are already in place in hospitals, although maybe unsystematic and heterogeneous. A more systematic and shared system would, in our opinion, better ensure equal access to technologies in all areas.

## Abbreviations

AdHopHTA: Adopting Hospital Based Health Technology Assessment
Finohta: Finnish Office for Health Technology Assessment
HB-HTA: Hospital-based HTA
HTA: Health technology assessment
MUMM: Managed Uptake of Medical Methods
